# Sensitization Potential of the Major Soybean Allergen Gly m 4 and Its Cross-Reactivity with the Birch Pollen Allergen Bet v 1

**DOI:** 10.3390/ijms26072932

**Published:** 2025-03-24

**Authors:** Ekaterina I. Finkina, Yulia D. Danilova, Daria N. Melnikova, Tatiana V. Ovchinnikova, Ivan V. Bogdanov

**Affiliations:** M.M. Shemyakin and Yu.A. Ovchinnikov Institute of Bioorganic Chemistry, Russian Academy of Sciences, 117997 Moscow, Russiaovch@ibch.ru (T.V.O.); contraton@mail.ru (I.V.B.)

**Keywords:** PR-10 allergens, birch Bet v 1, soybean Gly m 4, cross-reactivity, allergic sensitization, alarmins, lipid ligand, immunotherapy

## Abstract

The birch pollen allergen Bet v 1 is believed to be the main sensitizer among PR-10 allergens. Recent data have shown that some other PR-10 allergens also display sensitization activities, and Bet v 1-based immunotherapy is not effective for blocking allergic reactions to PR-10 proteins with low similarities to Bet v 1. Here, we investigated the sensitization potential of the major soybean allergen Gly m 4 and its cross-reactivity with Bet v 1. We demonstrated that Gly m 4 bound cholesterol and bile acids, including deoxycholic acid (DCA). Using qPCR, we showed that Gly m 4 induced the expression of genes encoding alarmins TSLP and IL-33 in intestinal-like Caco-2 cells; however, its fragments resulting from digestion by gastroduodenal enzymes or the DCA-bound Gly m 4 caused more pronounced gene upregulation. Using competitive ELISA, we demonstrated the low cross-reactivity of anti-Gly m 4 and anti-Bet v 1 IgG, raised in laboratory animals. Using mice allergy models with sensitization to birch or soybean allergens, we also showed a low cross-reactivity of Gly m 4- and Bet v 1-specific IgE, IgG1 and IgG2a. Thus, our findings support an assumption of the intrinsic sensitization capacity of Gly m 4 and the existence of Gly m 4-specific antibodies in sera of allergic patients.

## 1. Introduction

The prevalence of allergies has manifested a significant rise worldwide over the last few decades, currently affecting more than one billion patients and, thus, making allergic diseases an urgent health challenge [1]. In 2050, it is predicted that up to 4 billion people in the world will suffer from allergic diseases, including atopic dermatitis, allergic rhinitis and asthma [1].

PR-10 proteins constitute one of the most clinically relevant classes of plant allergens [2]. They are characterized by a wide distribution throughout the plant kingdom, have homologous regions in their structure, and, therefore, represent a class of pan-allergens responsible for pollen-food allergy syndrome due to the cross-reaction between pollen and food homologous PR-10 allergens [3]. Among PR-10 allergens, birch pollen Bet v 1 is one of the most clinically relevant and well-studied allergens. It was previously believed that with few exceptions, such as hazelnut Cor a 1.04 and carrot Dau c 1 able to induce sensitization, most PR-10 allergens could only elicit allergic reactions in Bet v 1-sensitized patients due to IgE cross-reactivity [4]. However, this paradigm is under reconsideration nowadays as new evidence about the intrinsic sensitizing capacity of other PR-10 allergens is arising.

**Figure 1:**
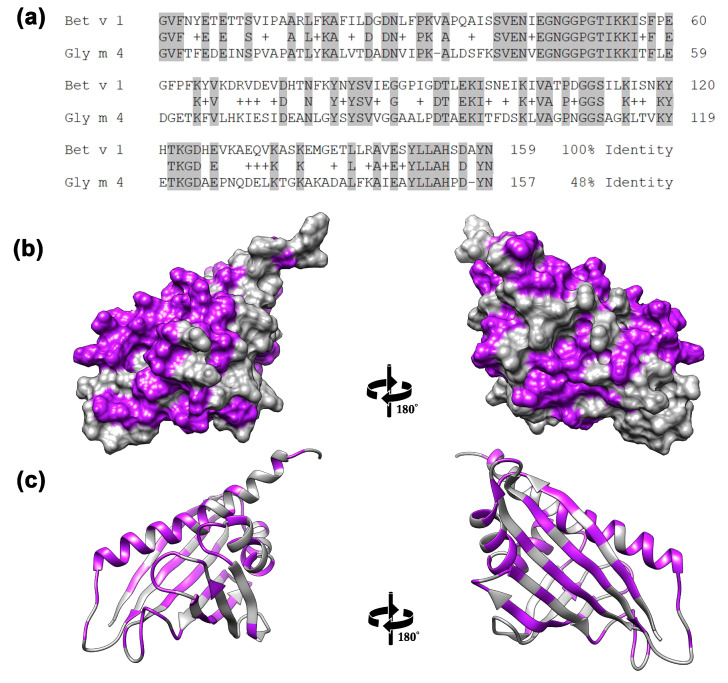
Comparison of soybean Gly m 4 and birch Bet v 1 structures. (**a**) Alignment of aa sequences of the allergens. (**b**) Surface and cartoon (**c**) representations of the structure of Bet v 1, in which the residues that match or differ in the structure of Gly m 4 are shown in grey or purple, respectively.

Gly m 4 is one of the most relevant soybean allergens, belonging to PR-10 proteins. This allergen cross-reacts with the birch Bet v 1 and is responsible for pollen-related soy allergy in Central and Northern Europe [5]. Apart from a few slight conformational differences, the three-dimensional structure of Gly m 4 is almost identical with that of Bet v 1. At the same time, the low sequence identity (48%) is characterized by the structures of these two allergens (Figure 1). As opposed to many other PR-10 food allergens, Gly m 4 is able to cause severe allergic reactions, including anaphylaxis after the consumption of soy-containing food, making this allergen a marker for severe food-allergic reactions to soy [6,7]. At the same time, the intrinsic sensitizing capacity of the soybean allergen Gly m 4 requires proof. This question is of great importance, because pollen extract is commonly prescribed for allergen-specific immunotherapy for patients with proved sensitization to birch pollen and related pollen-food allergies. However, a number of recent studies have shown the presence of IgE antibodies specific to allergens other than Bet v 1 in such patients and, thus, do not cross-react with Bet v 1 [8,9]. For such patients, immunotherapy with recombinant Bet v 1 or its hypoallergenic variants has been ineffective. For instance, clinical trials of sublingual immunotherapy (SLIT) for patients with birch-related apple allergy were not effective, and apple-induced oral allergy syndrome was not significantly reduced, because Mal d 1-specific IgE and IgG4 levels and Mal d 1-induced T-cell proliferation did not change significantly upon immunotherapy with birch pollen extract [10]. It has been also shown that double-blind placebo-controlled allergen immunotherapy by hypoallergenic Bet v 1 variant for patients with combined Bet v 1 and Gly m 4 sensitization failed to reach statistical significance in decreasing Gly m 4-specific IgE [11].

Due to the limited effectiveness of immunotherapy with birch pollen extract and Bet v 1 to treat birch-related food allergy, the main goal of this study was to investigate the sensitization potential of the soybean allergen Gly m 4 and its cross-reactivity with birch pollen Bet v 1. For this, the effects of Gly m 4 and its hydrolysates after processing by gastroduodenal enzymes on the expression of alarmin genes in intestinal-like epithelial cells Caco-2 were examined using qPCR. The impact of such a lipid ligand as deoxycholic acid (DCA) on the sensitization potential of soybean Gly m 4 was also shown. The cross-reactivity of specific Bet v 1 and Gly m 4 IgE from the sera of allergic patients as well as animal polyclonal anti-Bet v 1 and anti-Gly m 4 IgG was investigated in competitive ELISA experiments. Moreover, the cross-reactivity of allergen-specific IgE, IgG1 and IgG2a from the sera of mice sensitized by Bet v 1 or Gly m 4 was investigated.

## 2. Results and Discussion

### 2.1. The Ability of Gly m 4 to Bind Cholesterol and Bile Acids

As shown, many major allergens are able to bind lipid ligands, in particular, group 2 mite allergens, PR-10 proteins, lipid transfer proteins (LTPs), 2S albumins, lipocalins and others [12]. House dust mite (HDM) allergens from different groups are considered as fatty acid/lipid binding proteins [13]. Birch Bet v 1 and other PR-10 allergens are able to bind a wide variety of hydrophobic ligands, including flavonoids, plant hormones, fatty acids and sterols. Lipid ligands could stabilize the structure of allergens and increase their resistance to proteolysis as well as have an impact on the development of the Th2 allergic response.

**Figure 2:**
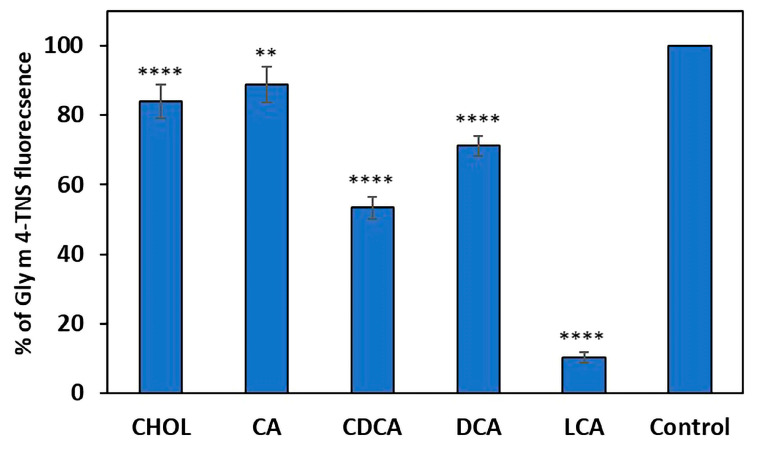
Effect of ligands on the fluorescence level of the Gly m 4-TNS complex. The results are expressed as the mean values (±SD) of the percentage of the fluorescence using the Gly m 4-TNS complex without ligand as a control. Significant differences (** *p* < 0.01, **** *p* < 0.0001) between sample means relative to control were analyzed by the *t*-test using GraphPad Prism.

We previously showed the ability of Gly m 4 to bind different fatty acids and lysophospholipids [14]. We assumed that, in certain conditions, the intact Gly m 4 might be able to reach the human intestine and form complexes with bile components, which can affect its allergenic potential. Therefore, here, we investigated Gly m 4 capacity to bind cholesterol (CHOL) and bile acids, which are the main components of human bile. Primary and secondary bile acids, such as cholic (CA), chenodeoxycholic (CDCA), deoxycholic (DCA), lithocholic (LCA) acids, were tested. The binding was studied by TNS displacement assay, when the testing ligand displaces fluorescent TNS probe from protein’s hydrophobic cavity and reduces its fluorescence. Gly m 4 bound LCA with high efficiency (10% of the control fluorescence) and less efficiently bound CHOL, CA, CDCA and DCA (83%, 88%, 53% and 71% of the control fluorescence, respectively) (Figure 2).

Thus, Gly m 4 bound to CHOL and all bile acids tested but with different efficiency. The composition of the intestinal bile acid pool is primarily characterized by 30% CA, 40% CDCA and 20–30% DCA, and only less than 5% LCA, which is characterized by high toxicity [15]. Previously, it was shown that Bet v 1 binds to sodium deoxycholate (DOC) and this ligand influenced allergenic properties of Bet v 1. DOC-bound Bet v 1 activated allergen-induced degranulation of basophils more efficiently than apo-Bet v 1. However, Bet v 1 in combination with this ligand caused a less pronounced immune response in a mouse model of allergic sensitization [12]. Based on all these data, DСA, which is one of the most abundant bile acids, was selected as a possible ligand of Gly m 4 for subsequent experiments.

### 2.2. Effects of Gly m 4 on the Expression of Alarmin Genes in Epithelial Cells

It is well known that epithelial cells play key roles in integrating countless environmental signals, including allergens [16]. Allergen sensing leads to the release of epithelial alarmins, such as thymic stromal lymphopoietin (TSLP), interleukin-33 (IL-33), IL-25, and others, which provides the initiation of the type 2 immune response to allergens [16]. The ability of PR-10 allergens to stimulate the production of alarmins by epithelial cells has been minimally studied. It was shown that pollen allergens, alder Aln g 1 and birch Bet v 1, caused the induction of the expression of genes encoding TSLP and IL-33 in human airway epithelial cells, Calu-3 [17]. Here, we investigated the influence of food soybean allergen Gly m 4 and its hydrolysates obtained after cleavage, mimicking gastroduodenal digestion in vitro on the expression of alarmins genes in intestinal-like epithelial Caco-2 cells. For that, Gly m 4 was digested sequentially at gastric pH 2.0 or 5.0 and under pepsin loading of 1:20 or 1:2000, respectively, and then at intestinal pH 7.0 with the mixture of trypsin/α-chymotrypsin as previously described [18]. At gastric pH 2.0, Gly m 4 was completely degraded and its large proteolytic fragments (with molecular weight more than 10 kDa) were not observed under SDS-PAGE (Supporting Materials Figure S1). However, not only proteolytic fragments but also intact Gly m 4 were observed on the electropherogram at gastric pH 5.0, mimicking allergen digestion in infants as well as in adults after soy-based liquid food consumption (Supporting Materials Figure S1). Peach Pru p 3, the major allergen of the LTP class, which is known to have the ability to upregulate genes encoding TSLP, IL-33, and IL-25 in Caco-2 cells, was used for comparison [19]. Pru p 3 was characterized by high proteolytic stability, and an intact allergen was present in the hydrolysate obtained, even at gastric pH 2.0 (Supporting Materials Figure S1).

**Figure 3:**
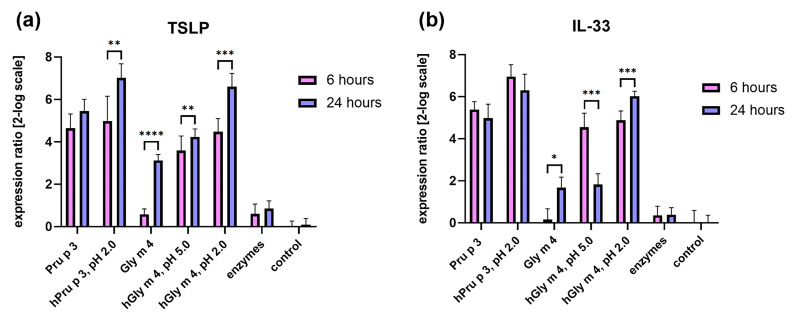
Influence of Gly m 4, Pru p 3 or their hydrolysates (hGly m 4 and hPru p 3) obtained after cleavage by gastroduodenal enzymes in vitro at gastric pH 2.0 or 5.0 on expression of alarmin genes in Caco-2 cells. Relative to control (untreated Caco-2 cells) expression of the genes encoding TSLP (**a**) and IL-33 (**b**) by epithelial cells after 6 and 24 h incubation with intact allergens or their hydrolysates is shown. The effects of gastroduodenal enzymes without allergens are also presented. Error bars represent standard deviation (±SD) between two biological and three technical replications for each biological one. Gene expression was compared with *t*-test using GraphPad Prism v.8.0.1. Significance levels for comparison of gene expression after 6 and 24 h of cell stimulation are: * *p* ≤ 0.05; ** *p* < 0.01; *** *p* < 0.001; **** *p* < 0.0001.

Pru p 3, the true food allergen, and its hydrolysate stimulated the expression of genes encoding TLSP and IL-33 with approximately the same efficiency, and this effect remained virtually unchanged over time (Figure 3). Intact Gly m 4 had a much less pronounced impact on the expression of these genes. At the same time, its hydrolysates, especially the one obtained at gastric pH 2.0, significantly increased the production of TSLP and IL-33, and this effect became more pronounced with increasing time from 6 to 24 h (Figure 3). Proteolytic enzymes without allergens had almost no stimulating effect.

Thus, the intact labile soybean allergen Gly m 4 is much less able to induce the upregulation of alarmin genes than the proteolysis-resistant peach Pru p 3. However, we showed that the proteolytic fragments of Gly m 4 are able to upregulate the genes encoding TSLP and IL-33, and this upregulation is much more pronounced compared to intact Gly m 4, which may play an important role in sensitization to the soybean allergen. Earlier, we showed that not only intact Gly m 4 but also some of its proteolytic fragments resulted from the digestion by gastrointestinal enzymes in vitro can selectively cross the Caco-2 polarized monolayer and induce the production of cytokines and chemokines responsible for the induction of the Th2 response by immune cells due to epithelial–immune cell communication [20]. This is why we proposed that one of the possible mechanisms of sensitization to soybean allergens might be mediated by proteolytic fragments of Gly m 4 that resulted after gastrointestinal digestion [20].

To date, there are limited data on the mechanisms by which epithelial cells sense allergens and regulate the production and release of alarmins. As shown, pattern recognition receptors (PRRs) are able to recognize allergens and promote allergic inflammation. For example, toll-like receptor 4 (TLR4) binds HDM, which leads to the activation of epithelial cells and production of TSLP, IL-25 and IL-33. Protease-activated receptors (PARs) are activated by proteases associated with many common allergens (e.g., HDM) or by mast cell tryptase and by trypsin released from damaged epithelial cells after allergen exposure, which leads to the release of IL-25 and TSLP. HDM, mold, cat, and cockroach allergens cause the activation of ripoptosome and maturation of IL-33 [13,21]. The mechanism for sensing PR-10 allergens by epithelial cells remains open.

As mentioned above, lipid ligands could affect the allergenic properties of the proteins and also themselves influence the development of the Th2 allergic response inducing the release of alarmins. For example, lipopolysaccharide (LPS) is a ligand of HDM allergens that plays an important role in HDM allergic inflammation [13]. Saturated but not polyunsaturated fatty acids promote allergic inflammation [13]. Bile acids derived from the intestinal microbiome metabolism of inulin fibers induce IL-33 production and influence type 2 immunity [22]. Here, we studied the effects of the possible ligand of Gly m 4, DCA, which is one of the most abundant bile acids, on the expression of alarmin genes in Caco-2 cells. Bet v 1, the major allergen of birch pollen, whose allergenic potential was shown to be influenced by DOC [12], was conditionally used for comparison in this experiment.

**Figure 4:**
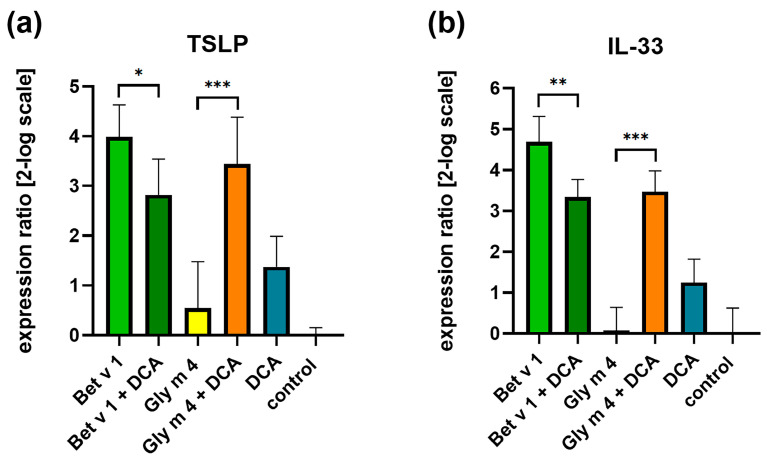
Impact of Gly m 4 and Bet v 1 in the presence or without lipid ligand DCA on expression of alarmins genes in Caco-2 cells. Relative to control (untreated Caco-2 cells) expression of genes encoding TSLP (**a**) and IL-33 (**b**) by epithelial cells after 6 h incubation with allergens in the presence or without DCA. Error bars represent standard deviation (±SD) between two biological and three technical replications. Gene expression was compared with *t*-test using GraphPad Prism v.8.0.1. Significance levels for comparison of gene expression after cell stimulation by allergens without or in the presence of DCA are: * *p* ≤ 0.05; ** *p* < 0.01; *** *p* < 0.001.

DCA and Gly m 4 induced weak upregulation of the genes encoding TSLP and IL-33. Bet v 1 stimulated the expression of both alarmins genes much more effectively than apo-Gly m 4. However, in the presence of the DСA, the stimulating effect was decreased in the case of Bet v 1 but increased in the case of Gly m 4 (Figure 4). In view of the opposite effect of the ligand on the properties of PR-10 allergens, we hypothesized that the formation of a complex with DCA may increase the ability of Gly m 4 but not Bet v 1 to sensitize. The observed effect may be due to the low similarity of Gly m 4 and Bet v 1; however, additional experiments are required to clarify this issue.

### 2.3. Cross-Reactivity of IgE from Sera of Allergic Patients

**Figure 5:**
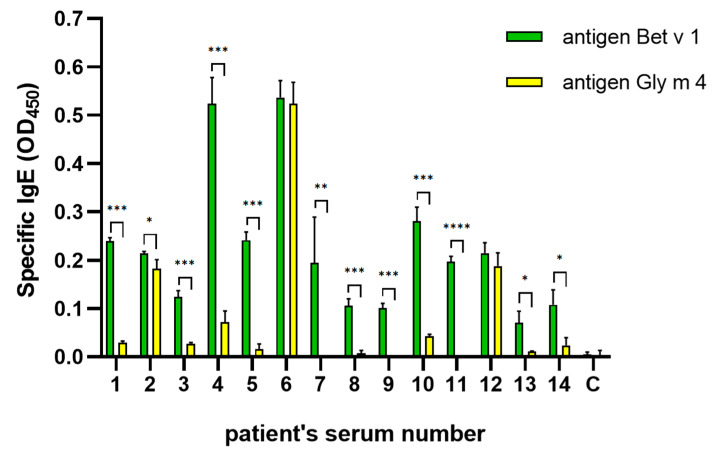
Allergen-specific IgE titers in sera of allergic patients determined by ELISA. C—negative control (serum of non-allergic patient). Standard deviations between three technical replications are shown as error bars. The *t*-test was used to compare OD_450_ values between Bet v 1 and Gly m 4 for each patient (significance levels are: * *p* < 0.05, ** *p* < 0.01, *** *p* < 0.001, **** *p* < 0.0001).

**Figure 6:**
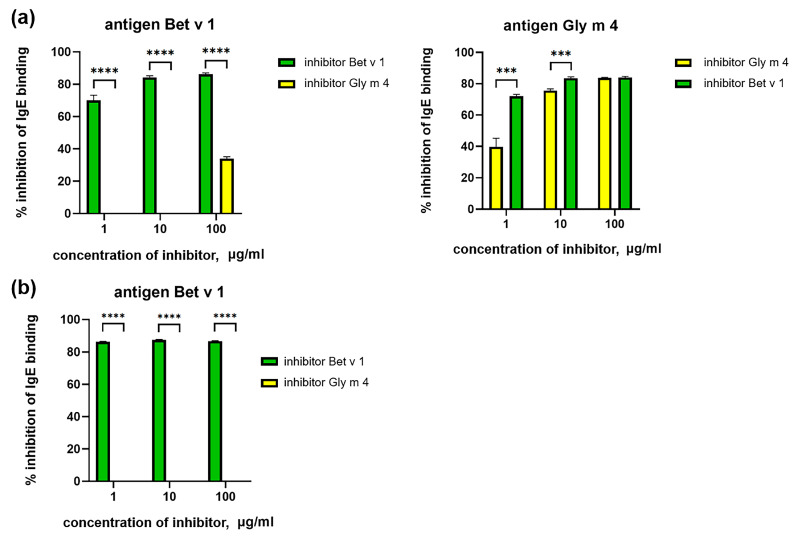
ELISA inhibition assay of binding of sIgE from sera of allergic patients #1 (**a**) and #7 (**b**). Bet v 1 and Gly m 4 were used as inhibitors of sIgE binding in different concentrations. In the serum of allergic patient #1 sIgE for both Bet v 1 and Gly m 4 were present. In the serum of allergic patient #7 sIgE only for Bet v 1 were present, therefore inhibition of sIgE binding only with Bet v 1 was performed. Standard deviations between three technical replications are shown as error bars. The *t*-test was used to compare effectiveness of inhibition by Bet v 1 and Gly m 4 (significance levels are: *** *p* < 0.001, **** *p* < 0.0001).

In our work, we used sera from patients with pollen and pollen-food allergy from the Moscow region with a prevalence allergy to birch pollen and low soy consumption (Table 1). The presence of IgE specific to Bet v 1 and Gly m 4 in sera of allergic patients was verified by ELISA. It was shown that, as expected, sIgE to Bet v 1 are present in most sera tested, and some of them contain specific-to-Gly m 4 IgE (Figure 5). ELISA inhibition assay was performed to estimate the cross-reactivity of sIgE from the sera of allergic patients. We obtained different results for sera specific to both Bet v 1 and Gly m 4 IgE, and for sera containing only anti-Bet v 1 IgE. In the first case, the binding of IgE to Bet v 1 was effectively inhibited by Bet v 1 at all used concentrations, while Gly m 4 had a weak inhibiting effect only at the highest concentration (100 μg/mL). Interestingly, the binding of Gly m 4 to IgE was effectively inhibited by both allergens, but Bet v 1 was more effective at low concentrations (Figure 6a, shown on the example of serum #1). In the second case, the binding of IgE to Bet v 1 was effectively inhibited only by Bet v 1 (Figure 6b, shown on the example of serum #7). All this, together, indicates that Bet v 1-specific or cross-reacting with both Bet v 1 and Gly m 4 IgE is present in the sera of allergic patients and that the sensitization to PR-10 proteins in the Moscow region is most likely with Bet v 1 and not with Gly m 4.

It has been demonstrated that cross-reactive Bet v 1 and Gly m 4 IgE are present in the sera of patients with birch pollen-soy allergy living in Central Europe, with a wide distribution of birch and other related trees, such as alder and hazel [5]. Moreover, an increase in allergic symptoms after soy consumption in Bet v 1-sensitized patients occurs during or even after the pollination season [23]. At the same time, birch pollen allergic patients often have specific-to-Gly m 4 IgE without reporting any symptoms to soy. A higher titer of Gly m 4-specific IgE is observed in patients with pollen-related allergy to soy, when they live in areas with a low prevalence of Betulaceae pollen and high soy consumption [24]. Thus, due to the ubiquity of birch and related trees, it is difficult to estimate the impact of sensitization to Gly m 4 in patients with allergic reactions to soy. In order to evaluate the cross-reactivity of antibodies of different classes, specific only to one of the allergens, including those involved in the development of allergic reactions, we further used laboratory animals with monoimmunization and monosensitization with birch Bet v 1 or soybean Gly m 4.

### 2.4. Cross-Reactivity of Animal Polyclonal Anti-Bet v 1 and Anti-Gly m 4 IgG

**Figure 7:**
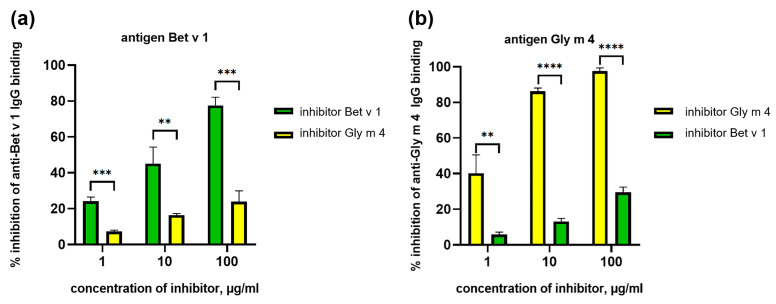
ELISA inhibition assay of the binding of animal anti-Bet v 1 (**a**) and anti-Gly m 4 (**b**) IgG. Bet v 1 and Gly m 4 were used as inhibitors of IgG binding in different concentrations in both cases. Standard deviations between three technical replications are shown as error bars. The *t*-test was used to compare effectiveness of inhibition by Bet v 1 and Gly m 4 (significance levels are: ** *p* < 0.01, *** *p* < 0.001, **** *p* < 0.0001).

First, the cross-reactivity obtained by animal immunization previously [4], polyclonal anti-Bet v 1 and anti-Gly m 4 IgG, was studied. It has been shown that polyclonal rabbit anti-Bet v 1 IgG or rat anti-Gly m 4 IgG bound Gly m 4 or Bet v 1 with less efficiency, respectively [4]. Сompetitive ELISA showed that the preincubation of immune antisera with the antigen used for the immunization significantly blocked the formation of an immune complex between IgG and the coated antigen (Figure 7a,b). At the same time, the use of the other allergens as an inhibitor (Bet v 1 in the case of anti-Gly m 4 IgG and Gly m 4 in the case of anti-Bet v 1 IgG) was much less effective in both cases, and for the inhibition of antibody binding, only 25–30% was observed in the highest concentration of the inhibitor (Figure 7a,b). The data obtained indicated low cross-reactivity of both anti-Gly m 4 and anti-Bet v 1 IgG, raised in animals immunized by a single allergen.

### 2.5. Animal Sensitization by Bet v 1 or Gly m 4

In the next step of this work, the cross-reactivity of mice antibodies, which were sensitized by Bet v 1 or Gly m 4, was examined. It is known that IgE-mediated allergy is characterized by skewing the Th1/Th2 response towards Th2 and rising allergen-specific CD4+ Th2 cells [25]. These T-cells are responsible for the production of Th2 cytokines (IL-4 and IL-13) and subsequent immunoglobulin switching for IgE production by B cells. In mice, allergen-specific IgE and IgG1 are commonly related to the Th2 response, while IgG2a antibodies are related to the Th1 response; this is why IgE and IgG1 antibodies represent good markers for the induction of an allergic response in mice [26]. It has been shown that bacterial endotoxins being exposed in mice together with allergens during the sensitization step induce decrease IgE and increase IgG2a levels, suggesting a switch in the immune response toward the Th1 profile [27]. This is why for the experiments with mice models of allergy, we decided to produce recombinant Bet v 1 and Gly m 4 allergens in an endotoxin-free ClearColi BL21(DE3) strain, having genetically modified lipopolysacchride (LPS), which is not able to induce toll-like receptor stimulation and subsequent NF-κB pathway activation [28].

**Figure 8:**
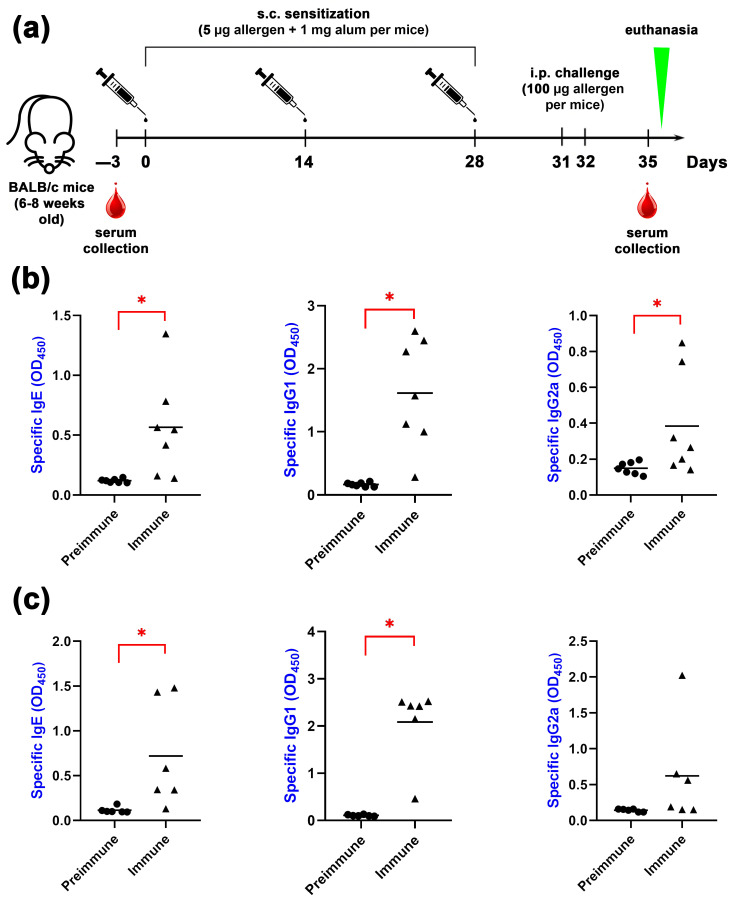
Schematic of the experimental procedures (**a**). 3 days before the first immunization (day-3), blood samples were collected from each mouse. Sensitization was performed three times at two-week intervals (on days 0, 14, 28). On days 31 and 32 mice were challenged with one intraperitoneal dose (100 μg of the corresponding allergen per mice). The blood samples were collected from each mouse on day 35; s.c.—subcutaneous, i.p.—intraperitoneal. Serum levels of IgE, IgG1 and IgG2a, specific to Bet v 1 (**b**) and Gly m 4 (**c**) before (preimmune) and after (immune) the sensitization. Statistical analysis was performed using the Wilcoxon matched-pairs signed-rank test and GraphPad Prism v.8.0.1 (GraphPad Software, La Jolla, CA, USA); significance level is: * *p* < 0.05.

Mice models of sensitization to pollen and food allergens are commonly used in order to study IgE-mediated allergic reactions [29]. Several allergic mice models to pollen Bet v 1 allergen are established and widely used due to the substantial significance of Bet v 1 in allergic research. The sensitization to Bet v 1 in mice models is usually developed by repeated subcutaneous or intraperitoneal injections of 1–10 μg of Bet v 1 adsorbed to 1–2 mg of Alum adjuvant per mouse, with varying numbers of injections and intervals between repeated immunizations. In the current study, we reproduced a previously established protocol for the development of mice model sensitization to Bet v 1 by three subcutaneous injections at two-week intervals (Figure 8a) [30]. In contrast to Bet v 1, there are no established allergic mice models to the soybean Gly m 4 allergen that have been published, which is why we applied the same sensitization protocol for the soybean Gly m 4 allergen as for Bet v 1. In order to assess the developed animal models, we evaluated the serum levels of allergen-specific IgE, IgG1 and IgG2a by ELISA (Figure 8b,c). We demonstrated that in both mice models of sensitization, allergen-specific IgE and IgG1 antibodies significantly (*p* < 0.05) increased in immune mice compared with preimmune ones (Figure 8b,c). It is interesting to note that in both mice models of sensitization, IgG2a was also increased after sensitization; however, only in the case of Bet v 1 was this rise statistically significant (*p* < 0.05).

Thus, for the first time, a mouse model of sensitization to the main soybean allergen, Gly m 4, was obtained, while, previously, mouse models of sensitization only to soybean extract had been established [31,32]. It is important to emphasize that the obtained mice model of sensitization with Gly m 4 has yet-limited clinical applicability, because it should be thoroughly evaluated with biological methods (basophil activation test, airway hyperreactivity test, evaluation of cytokine profiles, etc.). However, for the study of cross-reactivity between different classes of allergen-specific antibodies, this model was quite suitable, giving the results comparable with the model of sensitization with Bet v 1. In the two mouse models of sensitization with Bet v 1 and Gly m 4, the titers of specific IgE, IgG1 and IgG2a antibodies were quite similar.

### 2.6. Cross-Reactivity of IgG1, IgE and IgG2a from Sera of Sensitized Mice

**Figure 9:**
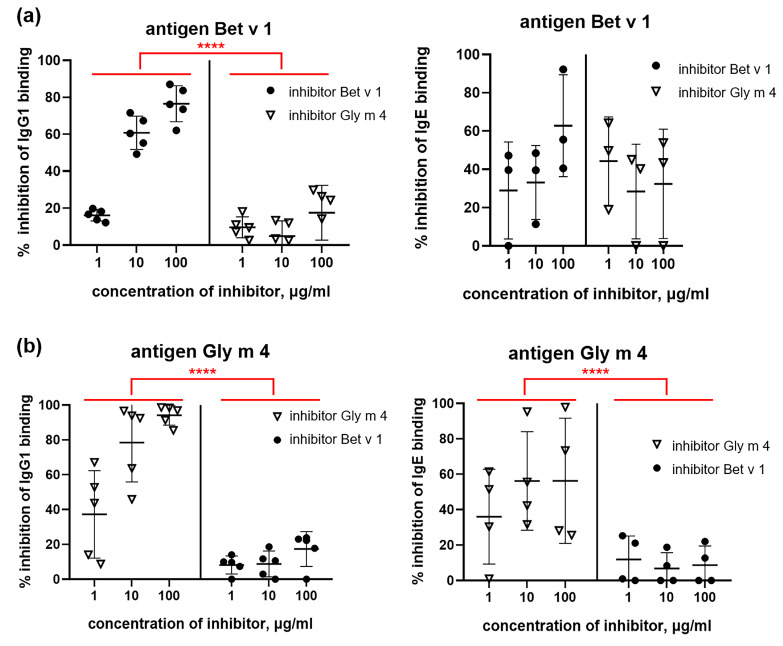
ELISA inhibition assay of the binding of specific IgG1 and IgE from sera of mice sensitized by Bet v 1 (**a**) or Gly m 4 (**b**). Bet v 1 and Gly m 4 were used as inhibitors of IgG1 and IgE binding in different concentrations in both cases. Statistical analysis was performed using Mann Whitney two-tailed test and GraphPad Prism v.8.0.1; significance level is: **** *p* < 0.0001.

Competitive ELISA was used for studying the cross-reactivity of specific IgG1 and IgE of mice sensitized by Bet v 1 or Gly m 4. It was shown that in the case of Bet v 1-sensitization, birch allergen itself inhibited the binding of specific IgG1 much more effectively (*p* < 0.0001) than Gly m 4. The difference in the capacity of Bet v 1 and Gly m 4 to inhibit IgE binding was much less pronounced and was not significant. A possible reason for this was the low levels IgE of specific to Bet v 1 in most mice sera (Figure 9a). For the group of mice sensitized by Gly m 4, the high specificity of IgG1 was also shown, and Gly m 4 itself inhibited the binding of these antibodies with soybean allergen much more effectively than Bet v 1 (*p* < 0.0001). In this mouse model, the binding of Gly m 4-specific IgE was also much more effectively inhibited by soybean allergen than by Bet v 1, and the difference was statistically significant (*p* < 0.0001). However, it is worth noting that a significant scatter of values was observed in this case (Figure 9b). Thus, our results indicated high specificity and low cross-reactivity of IgE and IgG1 from sera of mice sensitized by single Gly m 4 or Bet v 1 allergen.

**Figure 10:**
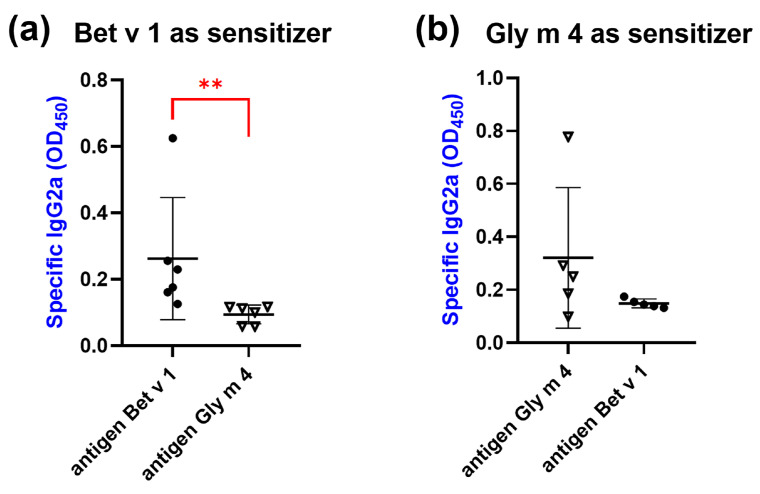
Bet v 1 and Gly m 4 binding of specific IgG2a from sera of mice sensitized by Bet v 1 (**a**) or Gly m 4 (**b**). Statistical analysis was performed using Mann Whitney two-tailed test and GraphPad Prism v.8.0.1; significance level is: ** *p* < 0.01.

The ability of blocking anti-Bet v 1 or anti-Gly m 4 IgG2a to bind both allergens was also tested. It was shown that anti-Bet v 1 IgG2a bound Gly m 4 to a much lesser extent than Bet v 1 (*p* = 0.0022) (Figure 10a). The binding of anti-Gly m 4 IgG2a with Bet v 1 was also lower than that for Gly m 4, but this difference was not statistically significant (Figure 10b). It is worth noting, however, that low titers of IgG2a were observed in the case of most mice in both models of sensitization; therefore, additional experiments are required to estimate the efficiency of the cross-blocking capacity of these antibodies to inhibit the development of cross-allergic reactions.

Thus, the results of all conducted experiments with laboratory animals show low cross-reactivity for different allergen-specific immunoglobulins. Taken together, the data obtained suggest the existence in the sera of patients with birch pollen and soy allergy not only cross-reactive but also antibodies specific to one of the allergens due to low sequence similarity between Bet v 1 and Gly m 4. This may particularly concern patients from regions with a low prevalence of Betulaceae trees and high soy consumption. The presence of Gly m 4-specific IgE, IgG1 or/and IgG4 classes may explain the inefficiency of immunotherapy with folding variant (FV) of Bet v 1 in patients with birch pollen-related allergy to soy [11]. Our data are in good agreement with the previously stated consideration that at least 80% of allergen homology is required to establish cross-blocking antibodies in immunotherapy-treated patients [33]. Nevertheless, it should be noted that the study of IgE cross-reactivity in ELISA assays has limitations and may not fully reflect the situation in vivo. Additional experiments using basophil or mast cell activation assays [34] would allow for a more complete characterization of the cross-reactivity of the studied allergens birch Bet v 1 and soybean Gly m 4.

## 3. Materials and Methods

### 3.1. Materials

Sera from patients (n = 40) with pollen and pollen-food allergy from the Moscow region with prevalence to birch were collected and kindly provided by the Clinical Diagnostic Center of the G.N. Gabrichevsky Research Institute for Epidemiology and Microbiology. RIDA qLine Allergy Panel 1–4 (R-Biopharm, Pfungstadt, Germany) was used to quantitatively determine the levels of specific IgE (sIgE) to allergen extracts in the patient sera. Using Enzyme-Linked Immuno-sorbent Assay (ELISA), sIgE to Bet v 1 were found in sera of 36 allergic patients and sIgE to Gly m 4 were also present in some of them; sera from 14 patients were used in subsequent experiments. Sera samples from non-allergic individuals were used as negative controls.

Colorectal adenocarcinoma Caco-2 cell line (ATCC HTB-37) was cultured in complete DMEM/F12 (1:1) (Gibco, Waltham, MA, USA) medium containing 10% fetal bovine serum (FBS, Capricorn Scientific, Ebsdorfergrund, Germany) and antibiotic-antimycotic solution (Invitrogen, Waltham, MA, USA) in a humidified CO_2_-incubator (5% CO_2_, 37 °C).

### 3.2. Recombinant Production of Allergens

Recombinant soybean Gly m 4 and birch Bet v 1 were obtained as described previously [20]. Histidine-tagged allergens His8-Gly m 4 and His8-Bet v 1 were obtained as follows. Plasmid vectors pET-His8-Bet v 1 (5907 bp) and pET-His8-Gly m 4 (5901 bp) were constructed by ligating previously obtained pET-His8 vector, created from the 5253 bp BglII/XhoI fragment of pET-31b(+) vector (Novagen, Merck Group, Darmstadt, Germany) and sequences of T7 promoter, ribosome binding site, lac-operator, and octahistidine tag, with sequences encoding Bet v 1 [GenBank X15877] and Gly m 4 [GenBank X60043]. His-tagged recombinant allergens were obtained by heterologous expression in *Escherichia coli* cells. For that, chemically competent ClearColi BL21(DE3) cells were transformed with pET-His8-Bet v 1 and pET-His8-Gly m 4 plasmid vectors by heat shock. ClearColi BL21(DE3)/pET-His8-Bet v 1 and ClearColi BL21(DE3)/pET-His8-Gly m 4 cells were inoculated in LB medium supplemented with 100 μg/mL ampicillin, 1 mM MgSO_4_, NPS (50 mM Na_2_HPO_4_, 50 mM KH_2_PO_4_, 25 mM (NH_4_)_2_SO_4_) and AI (0.5% glycerol, 0.05% D(+)-glucose, and 0.2% α-lactose monohydrate) solutions. The cells were grown at 37 °C for 24 h and were pelleted via centrifugation. His-tagged Bet v 1 and Gly m 4 were purified by metal chelate chromatography in the presence of 6 M Gu-HCl, dialysis and reversed-phase high-performance liquid chromatography on Reprosil-Pur C18-AQ (d 5 µm, 120 Å, 10 × 250 mm, Dr. Maisch GmbH, Ammerbuch, Germany) column in linear gradient of acetonitrile with 0.1% TFA (*v*/*v*). Homogeneity and the identity of the recombinant allergen samples were confirmed by SDS-PAGE, MALDI-TOF mass spectrometry and CD spectroscopy (Supporting Materials Figures S2–S4).

### 3.3. Ligand Binding Assay

A fluorescence-based ligand binding assay of Gly m 4 was carried out using TNS probe as described previously [17]. The assay was performed at 25 °C, with fluorescence measurements acquired at excitation and emission wavelengths of 320 nm and 437 nm, respectively, using an F-2710 spectrofluorimeter (Hitachi High Technologies America Inc., Pleasanton, CA, USA). TNS (4 µM) was incubated with or without bile acids (4 µM) for 1 min within a stirred cuvette in 10 mM phosphate buffer, pH 7.4. Following this initial incubation, the baseline fluorescence (F0) was recorded. Subsequently, Gly m 4 (4 µM) was added, and fluorescence was measured after a 2 min equilibration period (F). The results were expressed as a percentage of the fluorescence associated with the Gly m 4-TNS complex, calculated as [(F − F0)/FC × 100%], where FC represents the fluorescence of the Gly m 4-TNS complex in the absence of bile acids. All experiments were conducted in triplicates. Data were calculated using GraphPad Prism.

### 3.4. Stimulation of Caco-2 Cells

Caco-2 cells were seeded into the wells of 24-well plates in complete DMEM/F12 (1:1) medium containing 10% FBS and antibiotic-antimycotic solution and grown in a humidified CO_2_ incubator (5% CO_2_, 37 °C). Cells were grown over 15 days, with medium changing every 2–3 days. The formation of a cell monolayer was monitored using an inverted microscope (Olympus, Tokyo, Japan).

Firstly, Caco-2 cells were stimulated by adding fresh complete DMEM/F12 (1:1) medium containing intact Gly m 4 at a concentration of 4 µM or its hydrolysates obtained after soybean allergen cleavage, mimicking gastroduodenal digestion in vitro, as described [18]. Briefly, Gly m 4 was cleaved at pH 2.0 or 5.0 for 2 h at 37 °C using pepsin with enzyme-to-allergen mass ratio 1:20 or 1:2000, respectively, and further at pH 7.0 for 30 min at 37 °C using trypsin and α-chymotrypsin with enzyme-to-allergen mass ratio 1:400 or 1:100, respectively. The mixture was heated at 90 °C for 5 min to inactivate the enzymes. Pru p 3, a major peach food allergen of lipid transfer protein (LTP) class, at the same concentration and its gastroduodenal hydrolysate obtained by using pepsin digestion only at pH 2.0 were used for comparison. Digestion of Gly m 4 and Pru p 3 was monitored by SDS-PAGE (Supporting Materials Figure S1). The plates were incubated in a CO_2_ incubator at 37 °C for 6 or 24 h. Complete DMEM/F12 (1:1) medium or medium containing gastroduodenal enzymes without allergens was used in control wells.

Secondly, Caco-2 cells were stimulated with intact Gly m 4 or Bet v 1 at a final concentration of 4 µM as well as with these allergens in the presence of lipid ligand, deoxycholic acid (DCA), at a final concentration of 10 µM. The plates were incubated in a CO_2_ incubator at 37 °C for 6 h. Complete DMEM/F12 (1:1) medium or medium containing DCA without allergens was used in control wells. After incubation, the cells were washed with a PBS, lysed with ExtractRNA reagent (Evrogen, Moscow, Russia) and frozen at −70 °C.

### 3.5. RNA Extraction

Total RNA was isolated from the frozen samples according to the manufacturer’s instructions using ExtractRNA reagent (Evrogen). RNA purity and integrity were confirmed by measuring the A_260/280_ ratio (A_260/280_ values for all isolated RNA samples were above 1.8) using NanoPhotometer NP80 (Implen GmbH, München, Germany) and agarose gel electrophoresis (Supporting Materials Figure S5). RNase inhibitor (Evrogen) was added to final samples of total RNA, and 2 μg of total RNA was taken to synthesize the cDNA using MINT-Universal cDNA synthesis kit (Evrogen) containing MMLV-based reverse transcriptase following the manufacturer’s instructions. Two independent biological replicates for each condition were used.

### 3.6. qPCR

qPCR was performed on CFX Opus 96 Real-Time PCR System (Bio-Rad, Hercules, CA, USA) using the SYBR Green PCR kit (Evrogen) and forward/reverse target-specific primers: GGGGAGCCAAAAGGGTCATCATCT/GAGGGGCCATCCACAGTCTTCT for GAPDH (GeneBank: NM_02046, amplicon 235 bp) [35], AGGCCAACAGAGAGAAGATGACT/CGTCTCCAGAGTCCATGACA for actin-γ (GeneBank: X04098, amplicon 135 bp) [36], GCTATCTGGTGCCCAGGCTAT/CGACGCCACAATCCTTGTAAT for TSLP (GeneBank: XM_054353732, amplicon 131 bp) [37], CACCCCTCAAATGAATCAGG/GGAGCTCCACAGAGTGTTCC for IL-33 (GeneBank: XM_047424063, amplicon 115 bp) [38]. House-keeping genes encoding GAPDH and actin-γ were used as reference genes to normalize total RNA. The amplification was carried out using following thermal cycle profile: initial heating at 95 °C for 3 min, followed by 50 cycles of 95 °C—15 s, 62 °C—20 s, and 72 °C—30 s. The homogeneity of amplification products was assessed using agarose electrophoresis (Supporting Materials Figure S6) and melt curve analysis. Threshold cycle number (CT) of reactions was determined using the CFX Maestro software 2.0 (Bio-Rad, Hercules, CA, USA). PCR efficiency of 85–105% was confirmed by using LinRegPCR online tool (https://www.gear-genomics.com/rdml-tools/, accessed on 15 March 2025) [39]. The relative expression of the genes of interest was calculated by normalization to GAPDH and actin-γ using standard 2^−ΔΔCT^ method. Experiments were performed twice in three technical replications. The *p*-values ≤ 0.05 were considered significant.

### 3.7. Mice Models of Allergic Sensitization by Bet v 1 and Gly m 4

Female BALB/c mice at 6–8 weeks of age were purchased from Nursery for laboratory animals (Pushino, Russia) and kept under Specific Pathogen-Free (SPF) laboratory conditions in the Department of Experimental Biology with Vivarium of IBCh RAS (Moscow, Russia). Mice (n = 7 per Bet v 1 group and n = 6 per Gly m 4 group) were sensitized to His-tagged Bet v 1 or Gly m 4 by three subcutaneous injections of 5 μg of recombinant allergen adsorbed into 1 mg alum adjuvant (Alhydrogel adjuvant 2%, InvivoGen, San Diego, CA, USA) in a total volume of 150 μL of PBS on days 0, 14 and 28. On days 31 and 32, the mice were challenged intraperitoneally with 100 μg of Bet v 1 or Gly m 4 per mice. The control group (n = 3) was sensitized subcutaneously with PBS on the same days and challenged with PBS. Blood samples were taken on days −3 (preimmune) and 35 (immune) and were screened for Bet v 1- and Gly m 4-specific IgE, IgG1 and IgG2a by ELISA.

### 3.8. Immunoglobulin Binding Assay

For screening of human sIgE to Bet v 1 and Gly m 4 by ELISA, plate wells were coated with the recombinant allergens (0.5 μg/well) in PBS (pH 7.4) for 1 h at 37 °C. After blocking with 2% bovine serum albumin (BSA, SERVA, Heidelberg, Germany), an incubation with sera of allergic patients (in 1:4 dilution) was performed overnight at 4 °C. sIgE-binding was detected by using the HRP-conjugated goat anti-human IgE (1:2000 dilution, Sigma, St. Louis, MO, USA) and TMB liquid substrate, supersensitive for ELISA (Sigma). The enzymatic reaction was stopped by 2 N H_2_SO_4_, and absorbance values were recorded at 450 nm. Sera samples from nonallergic individuals were used as a negative control.

ELISA with polyclonal rabbit anti-Bet v 1 and rat anti-Gly m 4 antisera, obtained by repeated immunization of laboratory animals by Bet v 1 or Gly m 4 with Freund’s adjuvant [18], was performed as described above with some modifications. After blocking, the plate wells were incubated and diluted in PBS rabbit anti-Bet v 1 or rat anti-Gly m 4 antisera (from 1:400 to 1:291,600 serial dilutions) for 2 h at 37 °C. HRP-conjugated goat anti-rabbit IgG (1:50,000 dilution, Invitrogen, Waltham, MA, USA) and goat anti-rat IgG (Fine Test, Wuhan, Hubei, China) (1:6000 dilution in PBS with 0.5% BSA) were used as secondary antibodies.

Detection of Bet v 1- and Gly m 4-specific IgE, IgG1 and IgG2a from sera of sensitized mice was performed after blocking and overnight incubation at 4 °C of preimmune and immune animal sera, diluted in 1:10, 1:50 or 1:20 in PBS with 0.5% BSA, respectively. Biotinylated monoclonal anti-mouse IgE, IgG1 or IgG2a (in 1:2000, 1:1000 or 1:10,000 dilutions, respectively, Invitrogen) and HRP-conjugated streptavidin (in 1:10,000 dilution, Thermo Scientific, Rockford, IL, USA) in PBS with 0.5% BSA were used for the detection. Each experiment was carried out twice in three or four technical replicates.

### 3.9. ELISA Inhibition Assays

Competitive ELISA was performed for antibody-binding inhibition experiments. Diluted sera from allergic patients (in 1:4 dilution in PBS with 0.5% BSA), antisera from immunized animals (in 1:270,000 or 1:180,000 dilutions for rabbit anti-Bet v 1 or rat anti-Gly m 4 antisera, respectively) or sera from sensitized mice (in 1:100 or 1:50 dilutions for detection of IgG1 or IgE, respectively) were preincubated with Bet v 1 or Gly m 4 at concentrations of 1, 10 and 100 µg/mL for 1 h at 37 °C in order to inhibit antibody binding. Sera samples preincubated with PBS with 0.5% BSA were used as negative controls. The percentage of inhibition of antibody binding was calculated as: % inhibition = ((avODc − avOD)/avODc) × 100, where avODc—average OD_450_ of control wells; avOD—average OD_450_ of test samples. Each experiment was carried out twice in three technical replications.

## 4. Conclusions

The ability of PR-10 allergens other than birch pollen Bet v 1 to sensitize is an important issue, because this factor can affect the efficiency of allergen-specific immunotherapy by a single recombinant allergen. Here, we investigated the sensitization potential of the major soybean allergen Gly m 4 having only 48% of identity with Bet v 1. The cross-reactivity of soybean and birch PR-10 allergens was also studied.

We showed that Gly m 4 bound cholesterol and various bile acids, including DCA, which is one of the main components of human bile. We demonstrated that Gly m 4 was able to induce the expression of alarmins genes encoding TSLP and IL-33 in intestinal-like Caco-2 cells; however, its fragments after digestion by gastroduodenal enzymes or DCA-bound Gly m 4 caused more pronounced gene upregulation. By using sera from laboratory animals immunized with birch Bet v 1 or soybean Gly m 4 and sera from mice with allergic sensitization to one of these allergens, we showed the low cross-reactivity of Bet v 1 and Gly m 4-specific antibodies of different classes.

Thus, all the data obtained indirectly support the ability of soybean Gly m 4 to sensitize and the existence in sera of patients with birch pollen and soy allergy not only cross-reactive but also Bet v 1- and Gly m 4-specific antibodies. In summary, combination immunotherapy with both Bet v 1 and Gly m 4 or key epitope fragments of these two allergens might be effective in patients with pollen-food allergy, but further studies are required to confirm this assumption. Certainly, the identification of B-cell epitopes of Gly m 4, which, to date, have not been established yet, as well as the investigation of cellular cross-reactivity of major birch Bet v 1 and soybean Gly m 4 allergens, may shed light on the mechanisms of development for birch pollen-related allergy to soy.

## Figures and Tables

**Table 1:** Characterization of patient sera containing sIgE to Bet v 1 and Gly m 4.

No.	Age (y.o.)	Sex (M/F)	sIgE to Birch		sIgE to Soybean		**Another Plant Allergen Source**
			IU/mL	RAST Class	IU/mL	RAST Class	
1	4	M	>100	6	0.57	1.6	H, O, hz, pn, wn, po, cl, ct, wt, r, ss
2	31	M	>100	6	0.4	1.1	H, O, A, hz, pn, po, wn, cl, ct, wt, r, ss
3	5	M	>100	6	0.34	0.9	H, O, A, hz
4	6	M	>100	6	0.05	0.1	G, ct, po, wt, hz, pn
5	60	F	>100	6	-	-	A, H, hz, r, pn
6	4	M	>100	6	nd	nd	A, H
7	3	F	>100	6	nd	nd	A, H
8	9	M	>100	6	nd	nd	A, H, G, r
9	4	М	>100	6	-	-	G, ct, po, wt, hz, pn
10	10	F	>100	6	nd	nd	A, H, O, G, r
11	6	F	>100	6	-	-	A, H, G, pn, hz, ct, wt, r
12	6	F	>100	6	nd	nd	A, H, G, r
13	14	F	>100	6	nd	nd	A, H, O, G
14	3	М	13.44	3.7	-	-	wt, hz, pn

nd—not determined. Description of RAST classes: 0 class—not detectable or trace IgE level [0.00–0.34 IU/mL]; 1 class—threshold level [0.35–0.69 IU/mL]; 2 class—elevated IgE level [0.70–3.49 IU/mL]; 3 class—significantly elevated IgE level [3.50–17.49 IU/mL]; 4 class—high IgE level [17.5–49.99 IU/mL]; 5 class—very high IgE level [50.0–99.99 IU/mL]; 6 class—extremely high IgE level [≥100.0 IU/mL]. Inhalant: G = grass pollen, A = alder pollen, H = hazel pollen, O = oak pollen. Food: ct = carrot; po = potato; wt = wheat; hz = hazelnut; wn = walnut; cl = celery; pn = peanut; r = rye; ss = sesame.

## Data Availability

All data generated and analysed during this study are included in this published article and its Supplementary Materials.

## Supplementary Materials

The following supporting information can be downloaded at: https://www.mdpi.com/article/10.3390/ijms26072932/s1.
