# Supplementary material for: Sensitization Potential of the Major Soybean Allergen Gly m 4 and Its Cross-Reactivity with the Birch Pollen Allergen Bet v 1
Source: Int J Mol Sci. 2025 Mar 24;26(7):2932. doi: 10.3390/ijms26072932 (PMC11988912; doi:10.3390/ijms26072932)
Supplement: Supplementary file 1 [file ijms-26-02932-s001.zip › ijms-3492229-supplementary.pdf]

Supplementary information for

# Sensitization Potential of the Major Soybean Allergen Gly m 4 and Its Cross-Reactivity with the Birch Pollen Allergen Bet v 1

**Ekaterina I. Finkina *, Yulia D. Danilova, Daria N. Melnikova, Tatiana V. Ovchinnikova and Ivan V. Bogdanov**

M.M. Shemyakin and Yu.A. Ovchinnikov Institute of Bioorganic Chemistry, Russian Academy of Sciences, 117997 Moscow, Russia; danilova-julia2307001@mail.ru (Y.D.D.); d_n_m_@mail.ru (D.N.M.); ovch@ibch.ru (T.V.O.); contraton@mail.ru (I.V.B.)

* Correspondence: finkina@mail.ru; Tel.: +7-495-335-09-00

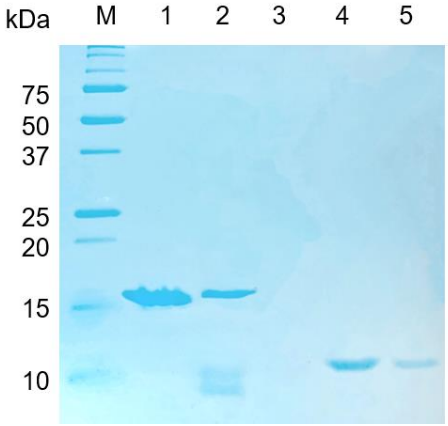


**Figure S1.** SDS-PAGE (15%T, 3%C in the separating gel) analysis of gastroduodenal digestion of Gly m 4 and Pru p 3 *in vitro* under different gastric conditions. 1 – intact Gly m 4; 2,3 – hydrolysates of Gly m 4, obtained at gastric pH 5.0 or pH 2.0, respectively; 4 – intact Pru p 3; 5 – hydrolysate of Pru p 3, obtained at gastric pH 2.0. M – molecular mass standards.

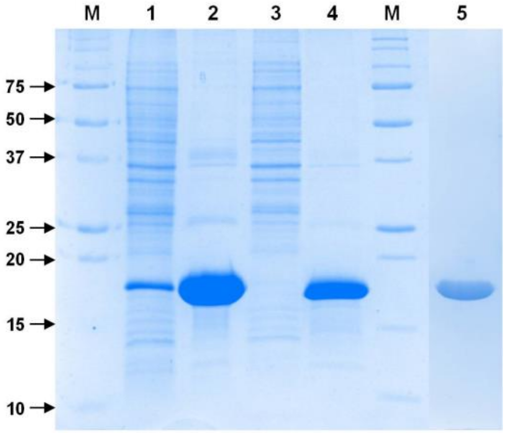


**Figure S2.** SDS-PAGE (15%T, 3%C in the separating gel) analysis of allergens purification. M — molecular weight marker; 1 — total *E. coli* cell lysate after His8-Gly m 4 expression; 2,3 — eluate containing His8-Gly m 4 and breakthrough fraction from IMAC column; 4,5 — His8-Gly m 4 or His8-Bet v 1 after dialysis.

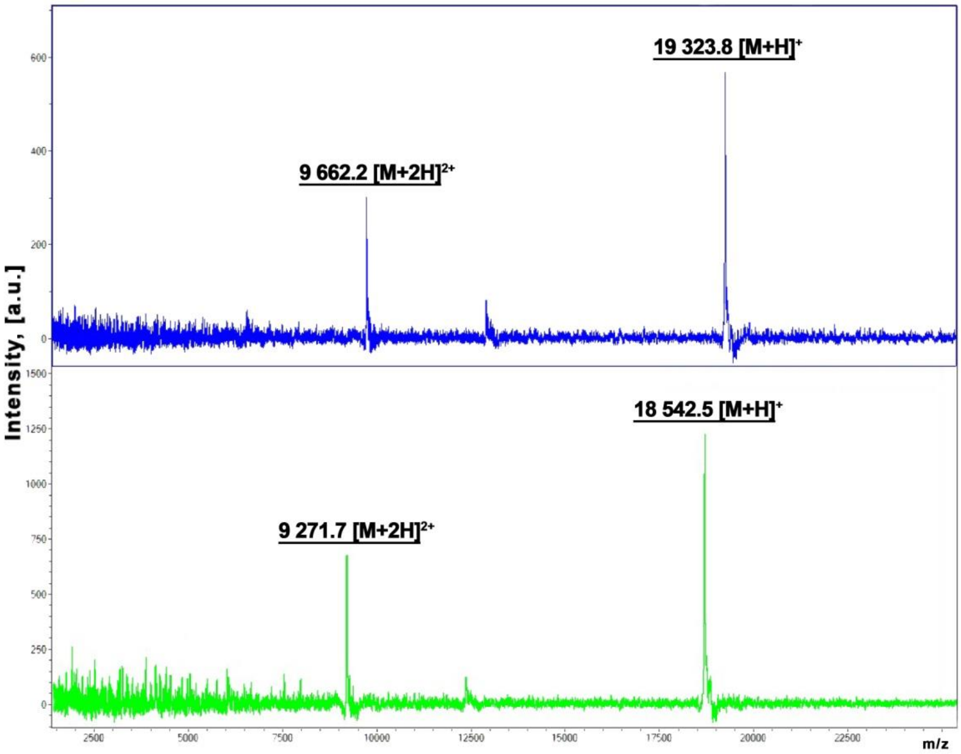


**Figure S3.** MALDI-TOF mass-spectra of recombinant His8-Bet v 1 (top) and His8-Gly m 4 (bottom). The calculated molecular weights of His-tagged Bet v 1 and Gly m 4 are 19 322,62 and 18 541,64 Da, correspondingly.

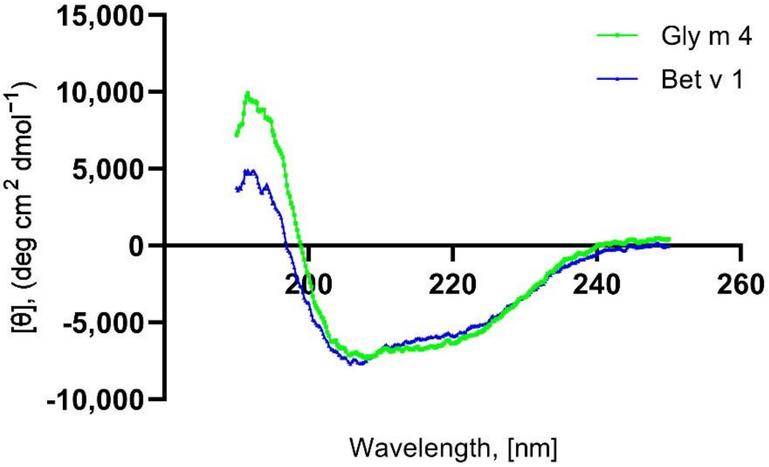


**Figure S4.** Circular dichroism (CD) spectra of His-tagged Gly m 4 and Bet v 1, recorded in PBS. Overlay of CD spectra of allergens reported as mean residue ellipticity on the y-axis as a function of wavelength in [nm] on the x-axis.

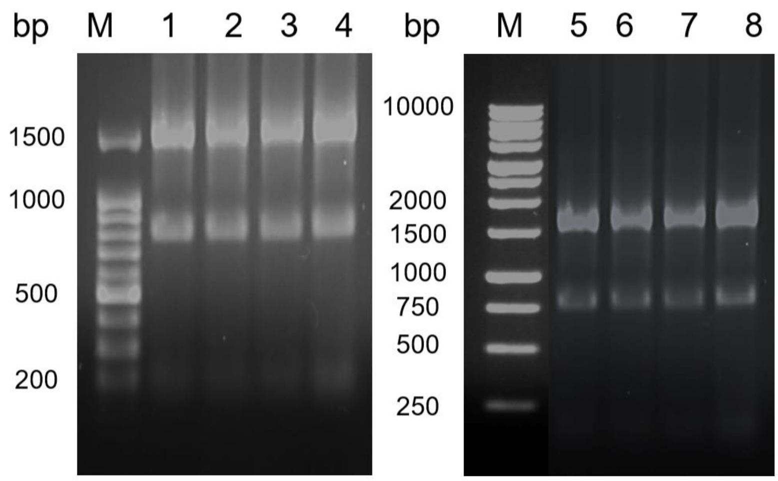


**Figure S5.** Examples of Caco-2 RNA quality as shown by 1.5% agarose electrophoresis. M – DNA ladder of varying length; 1-8 – total RNA from Caco-2 cells after incubation during 6 h: stimulated by Pru p 3 (1) or Gly m 4 (2); unstimulated control (3); stimulated by enzymes (4); stimulated by hydrolysates of Pru p 3 (5) or Gly m 4 (6), gastric pH 2.0; stimulated by hydrolysate of Gly m 4, gastric pH 5.0 (7); stimulated by Gly m 4 with DCA (8).

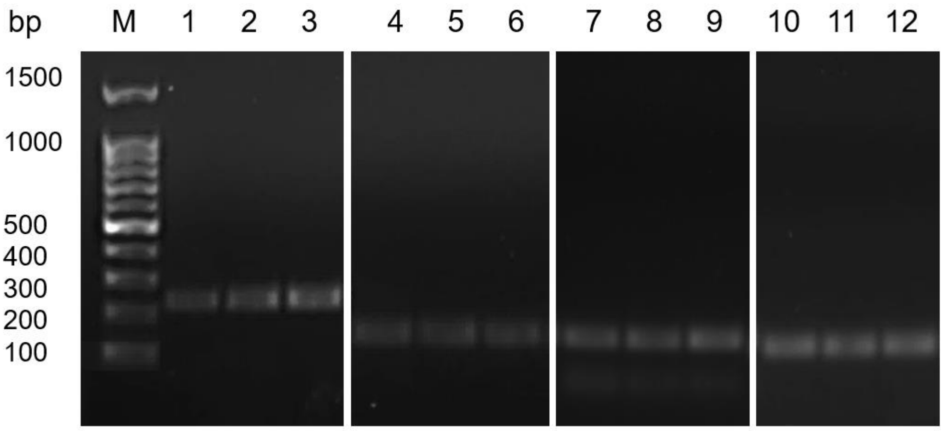


**Figure S6.** Agarose gel electrophoresis (1.5%) of qPCR products amplified using specific primer pair on *GAPDH* (1-3), *ACT-γ* (4-6), *TSLP* (7-9), *IL-33* (10-12) genes. M – DNA ladder of varying length.
